# Association of infertility and fertility treatment with mammographic density in a large screening-based cohort of women: a cross-sectional study

**DOI:** 10.1186/s13058-016-0693-5

**Published:** 2016-04-13

**Authors:** Frida E. Lundberg, Anna L. V. Johansson, Kenny Rodriguez-Wallberg, Judith S. Brand, Kamila Czene, Per Hall, Anastasia N. Iliadou

**Affiliations:** 1 Department of Medical Epidemiology and Biostatistics, Karolinska Institutet, Box 281, Stockholm, 171 77 Sweden; 2 Department of Oncology-Pathology, Karolinska Institutet and Reproductive Medicine, Karolinska University Hospital Huddinge, Stockholm, 141 86 Sweden

**Keywords:** Infertility, Ovarian stimulation, Gonadotropins, In vitro fertilization, IVF, ICSI, Mammographic density

## Abstract

**Background:**

Ovarian stimulation drugs, in particular hormonal agents used for controlled ovarian stimulation (COS) required to perform in vitro fertilization, increase estrogen and progesterone levels and have therefore been suspected to influence breast cancer risk. This study aims to investigate whether infertility and hormonal fertility treatment influences mammographic density, a strong hormone-responsive risk factor for breast cancer.

**Methods:**

Cross-sectional study including 43,313 women recruited to the Karolinska Mammography Project between 2010 and 2013. Among women who reported having had infertility, 1576 had gone through COS, 1429 had had hormonal stimulation without COS and 5958 had not received any hormonal fertility treatment. Percent and absolute mammographic densities were obtained using the volumetric method Volpara™. Associations with mammographic density were assessed using multivariable generalized linear models, estimating mean differences (MD) with 95 % confidence intervals (CI).

**Results:**

After multivariable adjustment, women with a history of infertility had 1.53 cm^3^ higher absolute dense volume compared to non-infertile women (95 % CI: 0.70 to 2.35). Among infertile women, only those who had gone through COS treatment had a higher absolute dense volume than those who had not received any hormone treatment (adjusted MD 3.22, 95 % CI: 1.10 to 5.33). No clear associations were observed between infertility, fertility treatment and percent volumetric density.

**Conclusions:**

Overall, women reporting infertility had more dense tissue in the breast. The higher absolute dense volume in women treated with COS may indicate a treatment effect, although part of the association might also be due to the underlying infertility. Continued monitoring of cancer risk in infertile women, especially those who undergo COS, is warranted.

**Electronic supplementary material:**

The online version of this article (doi:10.1186/s13058-016-0693-5) contains supplementary material, which is available to authorized users.

## Background

Infertility in couples has an estimated prevalence of 12 to 28 % [1]. Hormonal therapies are commonly used for treating a variety of infertility types [2]. For treatments of infertility aiming at inducing ovulation, clomiphene citrate or low-dose gonadotropins are usually given. For treatments involving in vitro fertilization (IVF) and intracytoplasmic sperm injection (ICSI), high doses of gonadotropins are required to stimulate multiple follicle recruitment (controlled ovarian stimulation, COS). Since all these treatments increase estrogen and progesterone levels, they have been suspected to influence breast cancer risk [3, 4]. So far, most studies and meta-analyses have found no clear association between ovarian stimulation and breast cancer risk [5–8]. However, many studies suffer from methodological limitations, including limited control for confounding factors, lack of an appropriate reference group, and short follow-up with small numbers of breast cancer cases among women using hormonal fertility treatment [5].

Mammographic density refers to the amount of radiologically dense fibroglandular tissue in the breast and is a major risk factor for breast cancer [9]. Women with extremely dense breasts have a four- to sixfold higher risk of developing breast cancer compared to women having fatty or non-dense breasts [10]. Mammographic density also shares many risk factors with breast cancer and is therefore seen as an intermediate in breast cancer etiology [11]. Like breast cancer, mammographic density is a hormone-responsive trait as it increases during hormone replacement therapy [12, 13], while it decreases with tamoxifen treatment [14, 15]. Since most women who have gone through fertility treatments are still below the age at which breast cancer is usually diagnosed, mammographic density is a useful marker to investigate the effect of hormonal fertility treatment on potential breast cancer risk.

Few studies have evaluated the effect of hormone stimulation for fertility treatments on mammographic density. A mammographic screening study showed no overall association between fertility drug use and mammographic density, although mammographic density appeared to be lower in women shortly after treatment initiation [16]. Apart from hormonal fertility treatment, the underlying infertility may also contribute to breast cancer risk. A recent study on women with primary infertility showed that ovulatory etiology of infertility was associated with a higher mammographic density, suggesting that these women may represent a group at high risk of breast cancer [17].

In the present study, we aim to investigate the associations between infertility, hormonal fertility treatments and mammographic density in a large screening-based cohort of Swedish women.

## Methods

### Study population

The KArolinska MAmmography project for risk prediction of breast cancer (KARMA) is a screening-based cohort study of women attending one of four mammography units in the national mammography screening program in Sweden between 2010 and 2013. In Sweden, women aged 40 to 74 years are offered mammography screening at an interval of 18 to 24 months. Each KARMA participant responded to a comprehensive web-based questionnaire covering information on age, education, anthropometry, reproductive health, lifestyle factors, medication, comorbidities and heredity. Raw and processed full-field digital mammograms were routinely collected at the screening visit and stored for further image processing. The KARMA cohort is also linked to the Prescribed Drug Register.

For the present study, we selected all women aged 40 to 69 years who had full-field digital mammograms stored at baseline (*n* = 57,481). Women with a previous malignant cancer (*n* = 5132) and women who reported breast surgery prior to mammography (*n* = 4364) were excluded. Women with missing information on fertility (*n* = 471), mammographic density (*n* = 82), parity (*n* = 206) and other covariate data (*n* = 3953) were also excluded, leaving a study population of 43,313 women for the analyses.

### Mammographic density measures

Mammographic density was measured from full-field digital mammograms collected at study entry using the fully automated system Volpara™ [18]. In brief, the algorithm computes the thickness of dense tissue at each individual pixel using the X-ray attenuation of an entirely fatty region as an internal reference. The absolute dense volume (cm^3^) is measured by integrating the dense thickness at each pixel over the whole mammogram, and the total breast volume (cm^3^) is derived by multiplying the breast area by the recorded breast thickness with an appropriate correction for the breast edge. The percent dense volume (%) is obtained from the ratio of these two measures and the absolute non-dense volume (cm^3^) by subtracting the absolute dense volume from the total breast volume. Volpara™ has been validated against breast magnetic resonance imaging data and we have previously shown that both percent and absolute dense volume area associated with established density determinants and breast cancer risk [19]. For analyses, we used the mean mammographic density from the left and right breast of the mediolateral oblique view.

### Exposure information

Information on infertility and fertility treatment was collected from the questionnaire administered at study entry. The participants who had ever tried to become pregnant for one year or more without success were defined as infertile, corresponding to the World Health Organization definition of infertility [20]. Women reporting a history of fertility problems were also asked if they had ever received fertility treatment and if so, which of the following: hormonal treatment only, sperm insemination, IVF/ICSI, IVF with egg donation, surgical treatment, and other treatment. Based on their answers, infertile women were further categorized into three exposure groups of hormonal fertility treatment; ever had COS for IVF or ICSI treatment (high-dose gonadotropin stimulation for multiple follicle recruitment and superovulation), ever had other hormonal treatment for ovulation induction (ovulation induction with clomiphene citrate or low-dose gonadotropins but no COS aimed at IVF/ICSI), and never had any hormonal treatment. The latter category included women who had not received any fertility treatment as well as women who had undergone a surgical treatment of infertility or insemination without ovulation induction. In Sweden, fertility treatments are provided within the tax-funded healthcare system, under the guidelines of the Swedish Society of Obstetrics and Gynecology, Special Interest Working Group on Fertility [21]. The treatments are individualized according to the infertility causes identified. For anovulatory infertility up to six treatment cycles using clomiphene citrate are usually prescribed as first-line treatment. If unsuccessful, low-dose gonadotropin stimulation treatments are initiated and thereafter IVF/ICSI. Intrauterine insemination alone or in combination with ovulation induction is indicated in cases of mild male factor or unexplained infertility. The Swedish tax-funded healthcare service covers up to six insemination treatments or up to three IVF/ICSI cycles. In general, if a couple does not achieve a pregnancy after three or four insemination treatments, the next step is using IVF/ICSI. If severe male factor, tubal factor, or other causes such as endometriosis are diagnosed, the appropriate first-line treatment is by using IVF/ICSI.

### Covariates

Information on the following covariates was retrieved from the questionnaire: age, height, weight, cigarette smoking, alcohol consumption, education level, family history of breast cancer, age at menarche, menstruation status, current use of hormone replacement therapy (HRT), and parity. Body mass index (BMI) was calculated from self-reported weight and height as kg/m^2^. Alcohol consumption was based on survey responses covering the frequency and amount of different alcoholic beverages consumed during the months before study entry, and calculated as mean intake in grams per day. Women who reported no drinking or drinking less than once per month were defined as non-drinkers. Family history of breast cancer was assessed for first-degree relatives (mother, full sisters, and daughters). HRT use was identified using the questionnaire and data from the Prescribed Drug Register. Women were defined as current HRT users if they reported using systemic HRT at study entry or, when questionnaire data was missing or incomplete, if they had any dispensation of systemic HRT within 100 days prior to mammography screening. Menopausal status was defined according to menstruation status, previous oophorectomy, and age. Women were considered postmenopausal if they reported not menstruating during the past year, had a history of oophorectomy, or were above the age of 55.

### Statistical analyses

We first compared mammographic density levels between fertile and infertile women overall. Next, we assessed associations between hormonal fertility treatments and mammographic density. For this, we used infertile women who had not received any hormonal fertility drugs as a reference, in order to prevent confounding by infertility per se. All associations were analyzed using generalized linear models (GLM) with a normal error distribution and a log link, to account for the skewed outcome distributions. We further applied robust standard errors using a sandwich estimator to account for additional under- or overdispersion and relax the assumption of log-normality. The GLMs yielded an intercept (β_0_) equal to the log mean density in the overall reference group and beta coefficients (β_i_) equal to the log mean ratios between exposed and unexposed groups. These were transformed to mean differences (MD) on the absolute scale:

$$ MD={e}^{\beta_0}\ast \left({e}^{\beta_i}-1\right) $$

The delta method was used to calculate variances and 95 % confidence intervals (CI) for the MD. Separate analyses were performed for absolute dense volume, absolute non-dense volume and percent dense volume. The mean differences are measured in cm^3^ for the absolute dense volumes, and percentage points (pp) for percent dense volume. All models were adjusted for age (5-year categories from 40 to 69 years). The fully adjusted models also included potential confounders categorized according to Table 1.

**Table 1:** Characteristics of the study population by history of infertility and fertility treatment

Characteristic	No infertility	History of infertility		
		No hormone treatment	Hormonal treatment only^a^	COS for IVF/ICSI^b^
	(*n* = 34,360)	(*n* = 5948)	(*n* = 1429)	(*n* = 1576)
Absolute dense volume, cm^3^	62.8 ± 33.2	64.0 ± 33.7	64.8 ± 31.4	73.8 ± 41.5
Absolute non-dense volume, cm^3^	773 ± 466	805 ± 470	804 ± 499	706 ± 465
Percent dense volume, pp	9.2 ± 5.2	9.0 ± 5.2	9.3 ± 5.2	11.4 ± 5.8
Age at mammography, years, %				
40–44	20.9	16.4	22.9	42.5
45–49	19.1	17.2	20.4	28.7
50–54	18.3	17.2	19.9	18.3
55–59	13.4	15.1	15.3	8.3
60–64	14.0	16.6	12.5	1.8
65–69	14.4	17.5	9.0	0.3
BMI, kg/m^2^, %				
<18.5	0.8	1.0	1.0	1.4
18.5–24.9	55.7	53.6	54.9	61.7
25–29.9	30.9	32.6	29.5	27.0
≥30	12.6	12.8	14.6	9.8
Age at menarche, years, %				
<12	12.6	14.2	14.1	16.1
12–14	72.0	70.5	69.7	70.7
≥15–25	15.3	15.3	16.2	13.2
Cigarette smoking, %				
Never	48.7	46.0	50.7	57.4
Current	11.3	10.8	9.2	7.2
Former	40.0	43.2	40.2	35.3
Alcohol consumption, g/d, %				
Non-drinker	18.3	17.3	17.4	15.5
0.1–9.9	62.1	61.2	62.8	67.8
≥10	19.5	21.5	19.9	16.7
Education level, %				
Compulsory school	10.9	12.0	6.9	2.5
Upper secondary school	31.6	31.1	32.4	25.5
University	54.3	53.1	56.8	69.2
Other	3.2	3.9	3.9	2.8
Family history of breast cancer, %	12.5	12.7	13.6	12.1
Parity, %				
Nulliparous	10.8	17.3	14.4	31.3
One birth	12.3	19.3	21.6	27.5
Two births	50.4	44.2	43.5	31.5
Three or more births	26.6	19.2	20.4	9.7
Premenopausal, %	46.1	39.3	50.2	73.9

Plus-minus values are means ± standard deviation

*Abbreviations: BMI* body mass index, *COS* controlled ovarian stimulation, *HRT* hormone replacement therapy, *ICSI* intracytoplasmic sperm injection, *IVF* in vitro fertilization, *pp* percentage points

^a^Hormonal treatment includes ovulation induction with clomiphene citrate or low-dose gonadotropins

^b^COS for IVF/ICSI treatment includes high-dose gonadotropin stimulation for multiple follicle recruitment and superovulation

The following sensitivity analyses were performed: first, we adjusted all models for age in finer intervals (1-year categories), to evaluate if there may be residual confounding by age in the main models. Second, we examined if the associations were modified by age, by estimating separate effects of the hormonal fertility treatments in ages 40–49 and ages 50–69. The effect modification was tested using the likelihood ratio test comparing models with and without interaction terms. Likewise, we tested for effect modification by parity (dichotomized). Third, we repeated the analyses excluding women who reported insemination, surgical or other non-hormonal fertility treatment (*n* = 493) from the group of infertile women having no hormonal treatment, keeping only women with untreated infertility in this group. Finally, we checked whether associations were different after excluding current users of hormone replacement therapy (*n* = 1899).

The significance level was 5 %, and all tests were two-sided.

SAS software (version 9.4, SAS Institute Inc., Cary, NC, USA) was used to prepare the data and Stata software (StataCorp. 2013. *Stata Statistical Software: Release 13.* College Station, TX: StataCorp LP.) was used for the statistical analyses.

### Ethics, consent and permissions

The study was approved by the Ethical Review Board at Karolinska Institutet, Stockholm, Sweden (ethical approval number 2010/958-31/1, amendment 2014/11-32). All participants provided written informed consent.

## Results

Characteristics of the study participants are summarized in Table 1, according to history of infertility and fertility treatments. Among all women, 8963 (20.7 %) reported a history of fertility problems. Of these, 1576 had gone through COS for IVF or ICSI, 1429 had had hormonal stimulation without COS and 5948 had received no hormonal fertility treatment. Infertile women with no hormone treatment, as well as those who had hormonal treatment without COS, had higher absolute dense and non-dense volume compared to non-infertile women, while percent dense volume was similar in these three groups. Women who had gone through COS treatments had higher absolute dense volume and lower non-dense volume, and thereby also a higher percent dense volume compared to the other groups. These women were younger and, consequently, more likely to be premenopausal compared to other infertile women as well as to women without fertility problems. They were also more likely to have a higher education level and a lower BMI. Infertile women who reported no hormonal treatment were older than women who reported fertility treatment. Nulliparity was more common in women who reported a history of infertility, with 31.3 % childless women in the COS-treated group.

**Fig. 1:**
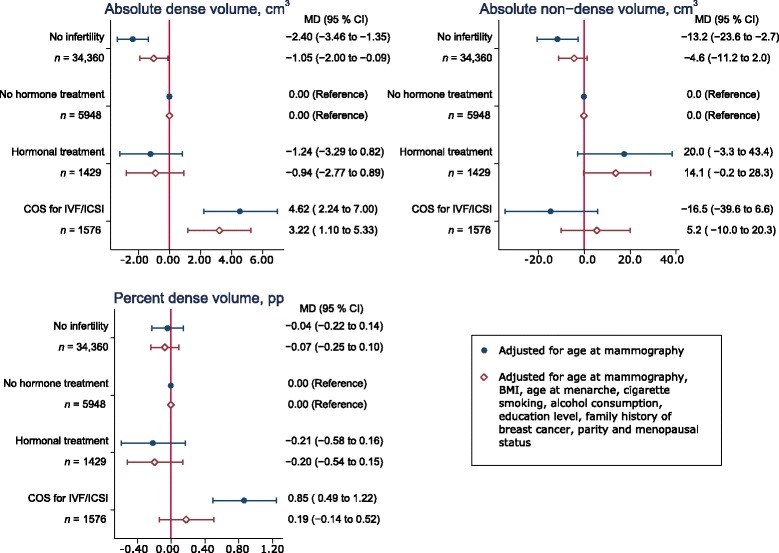
Association between infertility, fertility treatment and mammographic density. Legend: Hormonal treatment includes ovulation induction with clomiphene citrate or low-dose gonadotropins. COS for IVF/ICSI treatment includes high-dose gonadotropin stimulation for multiple follicle recruitment and superovulation. *Abbreviations: BMI* body mass index, *CI* confidence interval, *COS* controlled ovarian stimulation, *ICSI* intracytoplasmic sperm injection, *IVF* in vitro fertilization, *MD* mean difference (difference in mean density between exposed group and reference group), *pp* percentage points

The results of the models comparing women with and without a history of infertility are presented in Table 2. Figure 1 shows the results where infertile women were categorized by fertility treatment, and infertile women with no hormone treatment were the reference group.

**Table 2:** Association between history of infertility and mammographic density

		Age adjusted	Fully adjusted^b^
	N	MD (95 % CI)	MD (95 % CI)
Absolute dense volume, cm^3^			
No infertility	34,360	0.00 (Reference)	0.00 (Reference)
History of infertility^a^	8953	3.12 (2.22 to 4.02)	1.53 (0.70 to 2.35)
Absolute non-dense volume, cm^3^			
No infertility	34,360	0.0 (Reference)	0.0 (Reference)
History of infertility^a^	8953	13.8 (4.9 to 22.8)	7.6 (1.8 to 13.4)
Percent dense volume, pp			
No infertility	34,360	0.00 (Reference)	0.00 (Reference)
History of infertility^a^	8953	0.19 (0.04 to 0.35)	0.09 (−0.06 to 0.23)

*Abbreviations: BMI* body mass index, *CI* confidence interval, *MD* mean difference (difference in mean density between exposed group and reference group), *pp* percentage points

^a^All women who reported ever trying to conceive for at least 1 year without success

^b^Adjusted for age at mammography, BMI, age at menarche, cigarette smoking, alcohol consumption, education level, family history of breast cancer, parity and menopausal status

### Absolute dense volume

Overall, women with a history of infertility had 3.12 cm^3^ higher absolute dense volume (95 % CI: 2.22 to 4.02), compared to women without infertility (Table 2). After adjusting, the difference was 1.53 cm^3^ (95 % CI: 0.70 to 2.35). BMI and parity accounted for most of this attenuation. Compared to infertile women who had not had hormone treatment (Fig. 1), women treated with COS had on average 4.62 cm^3^ higher dense volume (95 % CI: 2.24 to 7.00). In the fully adjusted model, the difference was smaller (MD 3.22, 95 % CI: 1.10 to 5.33). We found no significant difference in dense volume between infertile women who had received hormonal treatment and those who had not (MD −0.94, 95 % CI: −2.77 to 0.89).

### Absolute non-dense volume

In the age-adjusted analysis, the absolute non-dense volume was 13.8 cm^3^ higher among infertile women than women without fertility problems (95 % CI: 4.9 to 22.8). In the fully adjusted model, infertile women still had a 7.6 cm^3^ higher absolute non-dense volume than women without fertility problems (95 % CI: 1.8 to 13.4). The attenuation was mainly driven by parity. When comparing to infertile women with no hormone treatment we found no significant differences, although non-dense volume seemed to be slightly larger in women with hormonal fertility treatment (MD 14.1, 95 % CI: −0.2 to 28.3).

### Percent dense volume

Percent dense volume was marginally higher among infertile women compared to women with no infertility (MD 0.19, 95 % CI: 0.04 to 0.35). After adjusting for possible confounders, the difference was no longer significant (MD 0.09, 95 % CI: −0.06 to 0.23). The attenuation was mainly due to adjusting for parity. Women who had gone through COS had higher percent dense volume than infertile women with no hormone treatment (MD 0.85, 95 % CI: 0.49 to 1.22), but this difference did not remain significant in the fully adjusted model (MD 0.19, 95 % CI: −0.14 to 0.52). No associations were observed with hormonal treatment other than COS in either age- or multivariable-adjusted analyses.

### Sensitivity analyses

In analyses stratified by age (Table 3), the association between COS and absolute dense volume seemed to be larger among 50–69-year-old women (MD 4.96, 95 % CI: 0.96 to 8.96) compared to women aged 40–49 years (MD 2.57, 95 % CI: −0.12 to 5.25). However, the likelihood ratio test for effect modification by age was not statistically significant (*p* = 0.107). Looking at percent dense volume on the other hand, a significant difference between the age groups was observed (*p* = 0.021). Compared to infertile women with no hormone treatment, percent dense volume was somewhat lower among women without infertility in the older, but not the younger age group. Due to the different age distributions of the exposure groups, adjustment for age was done with finer intervals (1-year categories), with similar results as in the main models (Additional file 1). The results from the effect modification models for parity are presented in Additional file 2. There was an indication that the association between absolute dense volume and COS treatment was stronger among nulliparous women (MD 7.33; 95 % CI: 3.29 to 11.38) than parous women (MD 2.21; 95 % CI: −0.02 to 4.44), with the *p* value for effect modification being close to significance (*p* = 0.073). The association with non-dense volume was modified by parity (*p* < 0.001), as a positive association between hormonal treatment other than COS was found in parous women only (MD 21.2, 95 % CI: 6.7 to 35.7) and not among nulliparous women (MD −24.7, 95 % CI: −61.8 to 12.4). Parity did not modify the associations with percent dense volume (*p* = 0.280). Excluding women with insemination, surgical or other fertility treatment from the group of infertile women with no hormone treatment gave similar results as in the whole study population (Additional file 3), as did excluding women who reported current use of hormone replacement therapy (Additional file 4).

**Table 3:** Effect modification by age on the association between infertility, fertility treatment and mammographic density

	Ages 40–49		Ages 50–69	
	N	MD (95 % CI)	N	MD (95 % CI)
Absolute dense volume, cm^3^				
No infertility	13,720	−1.08 (−2.74 to 0.59)	20,640	−1.05 (−2.15 to 0.04)
No hormone treatment	2000	0.00 (Reference)	5948	0.00 (Reference)
Hormonal treatment^a^	618	−2.81 (−5.63 to 0.01)	1429	0.93 (−1.47 to 3.34)
COS for IVF/ICSI^b^	1122	2.57 (−0.12 to 5.25)	1576	4.96 (0.96 to 8.96)
Absolute non-dense volume, cm^3^				
No infertility	13,720	−11.9 (−24.6 to 0.7)	20,640	−1.8 (−9.6 to 6.0)
No hormone treatment	2000	0.0 (Reference)	5948	0.0 (Reference)
Hormonal treatment^a^	618	6.9 (−17.6 to 31.4)	1429	17.2 (−0.9 to 35.2)
COS for IVF/ICSI^b^	1122	−3.5 (−23.9 to 16.8)	1576	11.7 (−13.7 to 37.1)
Percent dense volume, pp				
No infertility	13,720	0.10 (−0.16 to 0.36)	20,640	−0.26 (−0.48 to −0.04)
No hormone treatment	2000	0.00 (Reference)	5948	0.00 (Reference)
Hormonal treatment^a^	618	−0.12 (−0.60 to 0.35)	1429	−0.24 (−0.73 to 0.24)
COS for IVF/ICSI^b^	1122	0.23 (−0.17 to 0.64)	1576	0.48 (−0.16 to 1.11)

Models adjusted for age at mammography, BMI, age at menarche, cigarette smoking, alcohol consumption, education level, family history of breast cancer, parity and menopausal status. Each exposure category divided by age

*p* values for effect modification by age, using the likelihood ratio test; absolute dense volume: 0.107, absolute non-dense volume: 0.393, percent dense volume: 0.021

*Abbreviations: BMI* body mass index, *CI* confidence interval, *COS* controlled ovarian stimulation, *ICSI* intracytoplasmic sperm injection, *IVF* in vitro fertilization, *MD* mean difference (difference in mean density between exposed group and reference group), *pp* percentage points

^a^Hormonal treatment includes ovulation induction with clomiphene citrate or low-dose gonadotropins

^b^COS for IVF/ICSI includes high-dose gonadotropin stimulation for multiple follicle recruitment and superovulation

## Discussion

Women with a history of infertility had higher absolute dense and non-dense volume compared to non-infertile women. Among infertile women, those who had gone through COS treatment had higher absolute dense volume than those who had not received any hormone treatment. Hormonal treatment for ovulation induction without COS did not seem to be associated with either absolute or percent density, while non-dense volume was higher in this group.

Both absolute and percent dense volumes are associated with breast cancer risk [19]. The fibroglandular tissue in the breast, represented by the absolute dense volume, is considered the target tissue for tumor development [22], while percent dense volume incorporates additional information on the non-dense or fatty component of the breast. Percent dense volume is largely dependent on the absolute non-dense volume [23], explaining why observed differences in absolute dense volume do not necessarily translate to differences in percent dense volume [24–26]. In our study, infertile women had a higher absolute dense volume than women with no infertility. When comparing different hormonal fertility treatments, only the association between COS and absolute dense volume was significant compared to infertile women with no hormone treatment. In a study of breast cancer risk in relation to mammographic density among KARMA participants, absolute dense volume was associated with a higher risk of breast cancer [19]. In terms of effect size, the observed difference in absolute dense volume among women with COS (3 cm^3^) is comparable to the effect size previously reported for vigorous physical activity [23]. Within KARMA, this difference in absolute dense volume has also been linked to an approximately 2.5 % increase in relative breast cancer incidence [23].

To our knowledge, this is the first study addressing the impact of infertility and different hormonal fertility treatments on mammographic density, including COS. Observational data from Meggiorini et al. also pointed to high mammographic density levels among infertile women attending an IVF program [17]. This study, however, lacked a proper reference group and as such no strong conclusions could be drawn regarding the impact of female infertility. Another study by Sprague et al. found no difference in mammographic density between fertility drug users and non-users [16]. This study is not directly comparable to ours, due to the different density measure used and the lack of a reference group of infertile women. Moreover, this study did not address the impact of COS.

We found no association between hormonal fertility treatments other than COS and either absolute or percent dense volume, which is consistent with the results of Sprague et al. [16]. We did, however, observe a weak association between hormonal treatments other than COS and the non-dense volume, or amount of fatty tissue in the breast. This association was most evident in parous women, which is most likely explained by unmeasured differences between women with successful hormonal fertility treatments and women with fertility problems who eventually had a spontaneous pregnancy.

The findings of our study indicate that COS used for IVF/ICSI might have an effect on the breast tissue. Since the high doses of gonadotropins used in COS increase estrogen and progesterone levels to supra-physiological levels, it is possible that they have an indirect effect on the amount of dense tissue in the breast [3]. To our knowledge, there are no studies of long-term effects of COS on mammographic density. Our study lacked information on the timing of treatment, meaning we could not investigate if the higher density was limited to women who had recently received treatment. Nevertheless, in the analysis stratified by age, we found some indication of a stronger association in older compared to younger women, although the test for effect modification was not statistically significant. Although these results need to be interpreted with caution, they may indicate a potential long-term effect of COS on the breast tissue. While meta-analyses point to a null effect of IVF on breast cancer risk [5], more recent data seem to indicate a potential increase in breast cancer risk, which becomes more evident with increasing follow-up time [27]. Alternatively, the observed difference by age could also be explained by long-term effects of the underlying infertility, since infertility diagnosis may differ between infertile women who have gone through COS and those who have not.

A limitation of our study was the lack of information on the timing and number of treatment cycles each woman had gone through. We were also unable to differentiate between hormonal treatments with clomiphene citrate and low-dose gonadotropins. Assuming these treatments have different effects on the breast tissue, the estimates for hormonal treatment will be a mixture of these effects. Another possible limitation is the cross-sectional design, where mammographic density and history of infertility was assessed at the same visit. Since we relied on self-reports of infertility and fertility treatment there might be a risk of misclassification. However, the reporting of fertility problems is not likely to depend on the mammographic density and any potential misclassification should therefore be non-differential. Further, as infertility rarely requires in-patient care, we were unable to capture specific diagnoses of infertility through national registers. Hence, the group reporting a history of infertility will also include some non-infertile women who had an infertile partner. This would lead to an attenuation of the overall association between infertility and mammographic breast density. The infertility type could also influence what treatment the couples undergo, indicating that any association between treatment type and breast density could be due to the underlying infertility rather than the treatment per se.

The strengths of this study include the large, population-based design and the extensive background information collected on all study participants. Through the questionnaire we were able to identify women who had gone through fertility treatment but remained nulliparous, information that has been available in a national health quality register only for the last 8 years. We were also able to control for several important confounders. The quantitative volumetric method used for measuring mammographic density is fully automated and eliminates the issue of user variability in semi-automated methods.

## Conclusions

In this population-based sample of women attending mammographic screening, we found that women with a history of infertility had higher absolute dense volume than other women. Among the infertile women, those who had gone through COS had the highest absolute dense volume. This may indicate a potential adverse effect of COS, but could also be due to the underlying infertility. Whether this difference in density may affect their potential breast cancer risk is unknown. Hence, continued monitoring of women undergoing COS is warranted.

## Additional files

Additional file 1: Association between infertility, fertility treatment and mammographic density, adjusted by age in 1-year categories. (XLS 35 kb)
Additional file 2: Effect modification by parity on the association between infertility, fertility treatment and mammographic density. (XLS 35 kb)
Additional file 3: Association between infertility, fertility treatment and mammographic density, infertile women with non-hormonal fertility treatment excluded from reference group. (XLS 34 kb)
Additional file 4: Association between infertility, fertility treatment and mammographic density, current users of HRT excluded. (XLS 34 kb)

## Abbreviations

BMI: body mass index
CI: confidence interval
COS: controlled ovarian stimulation
GLM: generalized linear models
HRT: hormone replacement therapy
ICSI: intracytoplasmic sperm injection
IVF: in vitro fertilization
MD: mean difference
pp: percentage points
